# “To mean something to someone”: sport-for-development as a lever for social inclusion

**DOI:** 10.1186/s12939-019-1119-7

**Published:** 2020-01-14

**Authors:** Karen Van der Veken, Emelien Lauwerier, Sara Willems

**Affiliations:** 1 Department of Public Health & Primary Care, Research group Equity in Health Care, Ghent University, Ghent, Belgium; 2 Department of Experimental-Clinical & Health Psychology, Ghent University, Ghent, Belgium

**Keywords:** Sport-for-development, Social inclusion, Self-efficacy, Realist evaluation

## Abstract

**Background:**

Socially excluded groups are at higher risk of low well-being and poor health. The link between social exclusion and health inequities is complex, and not being involved in society makes it difficult to be reached by standard prevention programs. Sport-for-development (SFD) programs are low-threshold and may be promising settings for inclusive actions. We explore the underlying mechanisms through which SFD might have an impact on social inclusion and examine the necessary conditions that work as a catalyst for these underlying mechanisms.

**Methods:**

A realist evaluation approach was adopted. A non-profit SFD organization in a middle-large city in Flanders, Belgium, formed the setting for a single case study. Document analysis, participatory observations, interviews, and a focus group, were sources for identifying necessary context elements and essential mechanisms through which SFD could promote its participants’ health and wellbeing.

**Results:**

Among the most efficient mechanisms triggered by the Foundation’s activities are learning by fun, connecting with peers (of whom some serve as role model) and engaging as a volunteer with some responsibilities. Building trust in oneself and in others is a necessary process throughout all these mechanisms. Facilitating context factors include the activities’ accessibility and unconditional approach (creating a sense of safety), the popularity of the first division football team the Foundation is associated with (leading to a sense of belonging), a steady network of social partners and a strongly positive relationship with the SFD coach(es).

**Conclusions:**

Our findings demonstrate that a SFD setting may be a vehicle for engaging hard-to-reach population groups. It enhances socially vulnerable persons’ sense of competence and connectedness, leading to opportunities to improve life and work skills transferrable outside SFD settings. Based on these findings, suggestions are provided that may enhance the field and help to develop feasible (policy-led) interventions designed to promote social inclusion.

## Background

Social exclusion can be defined as the “lack or denial of resources, rights, goods and services, and the inability to participate in the normal relationships and activities, available to the majority of people in a society” [1]. It is inherently multi-causal and relational in nature, and leads among others to loss of status, autonomy, self-esteem, and expectations [2]. Socially excluded often find themselves in a downward spiral: inadequate access to food, housing and other basic resources, lead to adversity and poor health [3–5], further complicating the access to services that enhance the ability of the socially excluded to cope with their situation (e.g. education, sport and preventive health services, healthy life and work conditions…) [6]. Socially excluded youth, for example, is at higher risk of (chronic) health complaints, mental health problems and adult morbidity and mortality [5, 7–9]. Sport has the potential to increase individuals’ resilience, here defined as “the ability to adapt to adversity or to cope” or as “a reduced vulnerability for the adverse outcomes of stress or dysfunction” [10, 11]. A systematic review reported 40 different psychological and social benefits of participation in sport, with as most common positive outcomes fewer depressive symptoms, higher self-esteem, better social skills, higher confidence and higher competence amongst sport participants than non-sport participants [12]. Moreover, a healthy lifestyle including physical exercise is effective in preventing chronic diseases at a later age, especially when starting in childhood [13–16]. However, the abundant positive outcomes of sport participation need to be put in context. Regular sport clubs are not accessible to all. Especially for those at risk of social exclusion, participation in sport and leisure activities is limited, due to financial, geographical and socio-cultural barriers [17–19]. Yet, precisely at-risk persons could benefit most from both the health improving and resilience-enhancing effect of sport), for they encounter more health related problems [5, 8, 9]. Sport-for-Development (SFD) is a potential answer to the catch-22 of those needing it most not being able to access sport and benefit from it. SFD can be defined as “the use of sport to exert a positive influence on public health, the socialization of children, youths and adults, the social inclusion of the disadvantaged, the economic development of regions and states, and on fostering of intercultural exchange and conflict resolution” [20]. Socially vulnerable groups can be reached more easily by such locally organized, accessible initiatives in comparison to standard sport clubs, because (geographic, financial, cultural and social) barriers are lifted and because participants are actively recruited [21, 22]. SFD has increasingly been linked to positive outcomes such as personal development and enhanced resilience [22–33]. These may be important intermediate outcomes, and may further enhance chances on employment and other opportunities for social inclusion. Evidence has not only to be gathered regarding the outcomes of SFD with the aim of social inclusion, but also under which circumstances and how SFD may lead to its successes. Insight into to these practices, and, more specifically, what works for whom in which circumstances may provide valuable information for the design of (policy-led) interventions designed to combat social exclusion. The current study is part of a four-year (2016–2019) transdisciplinary research project – CATCH (*Community Sports for AT-risk youth: innovative strategies for promoting personal development, health and social CoHesion*) - aimed at the exploration of how and when low-threshold sport practices have their effect in promoting social inclusion. In the first phase of the CATCH research project, a program theory (PT) was developed on how and under which conditions low threshold sport practices may be a vehicle for social inclusion of socially vulnerable populations. This theory (cf. Table 1) was developed based on a multiple case study and insights from literature review.

**Table 1.**
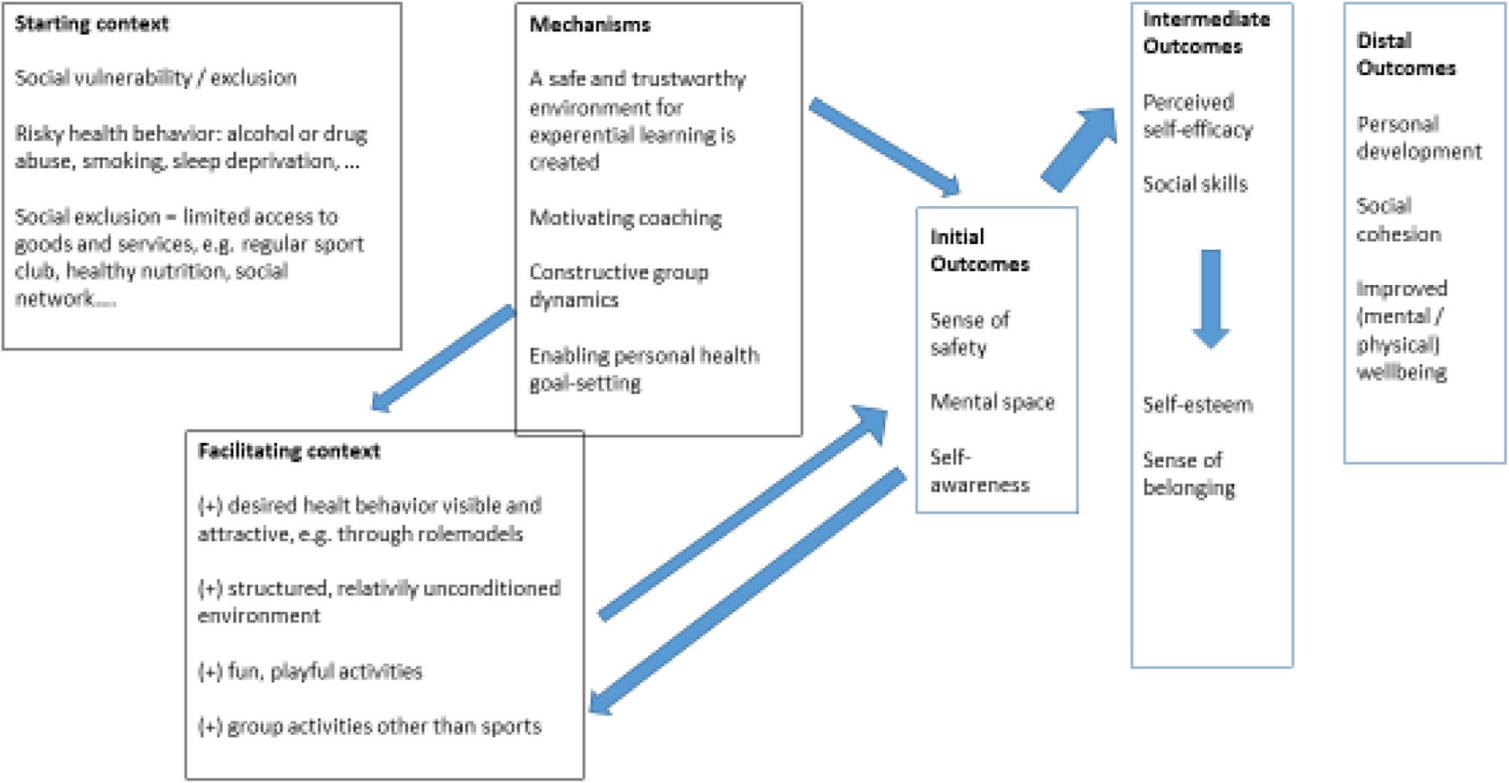
CATCH theory on SFD as lever for health and social inclusion

In the current study, we aim to test and refine this theory, through an evaluation of a middle-large SFD organization in Flanders, Belgium.

### Studied case

We evaluated activities of the KAA Gent Foundation (further referred to as ‘the Foundation’), the product of a public-private partnership between the city of Ghent and its first division football club KAA Gent which is located in Ghent, a middle-sized city in the northern part of Belgium (Flanders), at the edge of one of Flanders most deprived neighborhoods [34]. The Foundation embodies the football club’s social return to society in the form of activities generating social cohesion, health and inclusion, especially for vulnerable populations in Ghent and its surroundings [35]. In 2018, 566 persons participated in one of the 743 social emancipatory and sportive activities (25,409 contact hours with target population). The football teams GB and GP counted on average 15 participants in every training.

All community work of the Foundation is organized alongside their policy model, referred to as *#COBW* (Come on Blue White, referring to the colors of the club) and explained in Table 2.

**Table 2.**
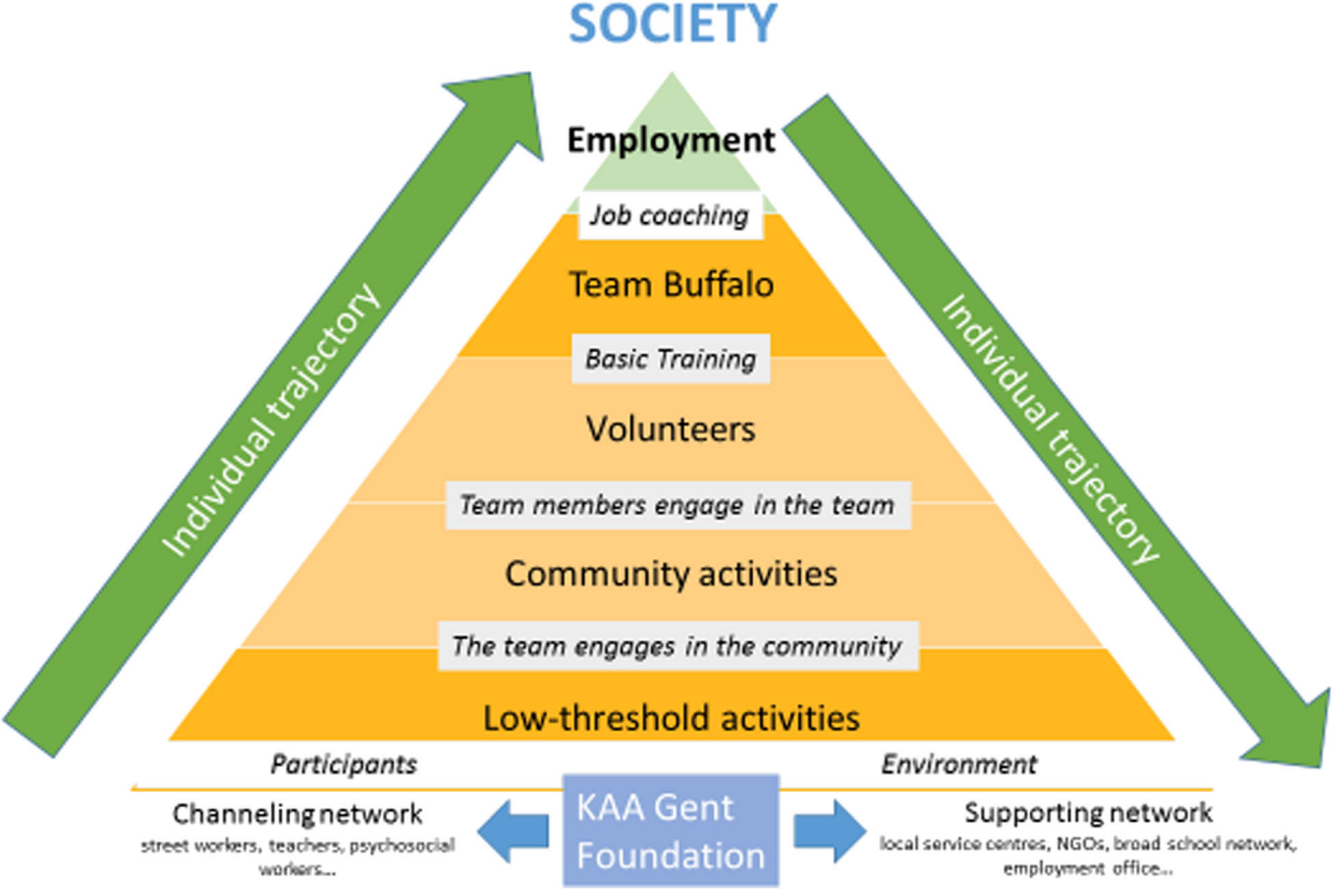
#COBW Policy model KAA Gent Foundation

The KAA Gent Foundation case study aims at examining 1) which conditions are put forward by the SFD organization in promoting social inclusion and appear to be necessary elements to have its effects; and 2) what mechanisms are found to exist and are perceived of as essential working elements to have an impact within the context of this particular SFD organization.

## Methods

### Design of the evaluation study

A realist evaluation (RE) was implemented [34], which aims at identifying the hidden causal forces behind empirically observable patterns or changes in those patterns [37]. This is done through ‘retroduction’: going back from observed patterns and looking below the surface for what might have produced them [38, 39]. Realist thinking thus starts from the empirical outcome, tracing processes backwards to study the question ‘What works for whom, why, and under which circumstances?’ [36] through identification of the key mechanisms (M), influential context factors (C) and expected outcomes (O). Context-Mechanism-Outcome (CMO) configurations then serve as a heuristic for theory development, clarifying what preceded the visible outcome. The output of a realist evaluation is a program theory (PT) or, as is the case in this study, a refined PT (that builds further / tests an already existing PT).

### Data collection & analysis

The case study of the KAA Gent Foundation took place between January and December 2018. During that time, a number of qualitative data were collected through, respectively, document analysis, observations of group activities, in-depth interviews and a focus group discussion (FGD). An overview of the data sources can be found in Additional file 1.

**Fig. 1:**
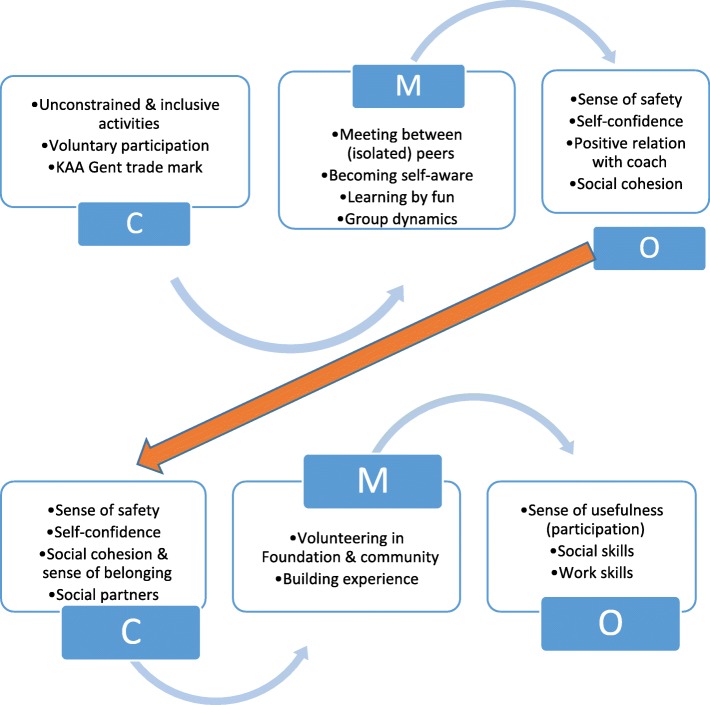
Program theory underpinning KAA Gent Foundation’s policy model

First, the main policy documents and reports of the foundation have been studied, of which the most important appeared to be the Foundation’s Strategic Policy Plan 2017–2020, in which the Foundation’s policy model is explained (cf. Table 2). Document analysis taking place before the interviews and FGD allowed the researchers to identify an implicit program theory (cf. Results - Fig. 1) underlying the Foundation’s policy model, and to consequently structure the interviews and FGD as such that the supposed mechanisms described in the underlying PT could be tested (i.e. confirmed, denied or adapted by interviewees). Other documents analyzed were: the Foundation’s two latest year reports (2017, 2018), its subvention policy showing how social return is required for subventions given to local football clubs, some chats of closed Facebook groups, the curriculum of the Team Buffalo socio-educative trainings and updates on the Foundation’s website. Document analysis mainly increased the understanding of how the Foundation defines ‘social inclusion’ into a couple of proxy indicators and provided insight in how the Foundation communicates with participants and stakeholders.

From May to July 2018, one to two researchers observed training activities (in a participatory way whenever possible), team events and tournaments, of which they took field notes in a semi-structured observation report, focusing on the identification of key mechanisms (M) and context (C) factors. In the data analysis, these elements were counterchecked with context, mechanisms and outcomes identified through interviews and FGD. The following subprojects were observed: Buffalo Dance Academy: a dance school for children aged 12–15 years in a deprived neighborhood near the stadium; Buffalo League: a series of community-based activities with children (2–12 years) from schools in the same deprived neighborhood; Geestige Buffalo’s (*Funny Buffalos*, further referred to as GB): a mixed (male + female) football team for adults (18+ years) with psychosocial and/or psychiatric difficulties; Gantoise Plantrekkers (‘*Astutes from Ghent’*, further referred to as GP): a separate male / female football team for socially deprived adults (18+ years), e.g. homeless or people struggling with addiction.

During the participatory observations, relations of trust have been established with participants, enabling in-depth interviews (October–November 2018) with eleven of them. Interviewees were selected based on their interest in telling their story, taking into account a fair distribution between the GB (*N* = 6) and the GP (*N* = 5), between habitués and newcomers, and a representation of the different vulnerabilities (poverty, homelessness, addiction, psychosocial difficulties…) faced by the participants. Recruitment of participants for key informant interviews took place during the participatory observation. Since women are underrepresented both in the GB and GP, this shows in the gender distribution of interviewees. For this semi-structured in-depth interview, with an average length of 50 min, an interview guide was used that allowed exploration of key mechanisms and context factors as identified in the Foundation’s PT (cf. Figure 1). Interviews were audio-recorded upon permission of the interviewee (9 out of 11) - when not audio-recorded, notes were made by the interviewer.

Lastly, a FGD (*N* = 8) took place (November 2018). Participants (two coordinators, 3 social partners, two participants of the Foundation’s activities and one SFD policy expert) were purposively selected. Having observed respectful and straightforward communication between these stakeholders for almost a year, we were confident this mixed constellation would not endanger any of the participants and might be an opportunity for open discussion and in-depth insights in the functioning of SFD in general, and the Foundation and its network in specific. The FGD was moderated by two experienced researchers in qualitative research and discussed the findings of the (anonymized) interviews, and parallels between the Foundation’s policy plan and a by the researchers developed PT (cf. Table 1) on how SFD may impact the participant’s health and wellbeing. The FGD was audio-recorded and transcribed.

All documents, observation reports, interview and FGD transcripts were entered in NVivo 11 software. Three researchers were involved in the data analysis. First a process of open, axial and selective coding was carried out separately by two researchers, with regular discussions to find common ground on their respective analysis of the data. Whenever conflicting analyses occurred, feedback was requested from key stakeholders or a third CATCH researcher. Then the research team developed hypotheses as to how and in which circumstances the Foundation’s work might lead, or not, to improved wellbeing. These hypotheses were profoundly studied, searching data actively for key mechanisms (M) generating intended and unintended outcomes (O) concerning the SFD participants’ wellbeing and for context factors (C) triggering or hindering these key mechanism. The hypotheses were also discussed in the FGD.

Written informed consent was taken from all participants. The study was approved by the ethical committee of Ghent University (number B670201836103).

## Results

This section consists of three parts. In the first part, we translate the Foundation’s policy model #COBW (cf. Table 2) into a realist program theory (Fig. 1). This analytic step preceded and inspired further data collection (interviews and focus groups). In a second part, we scan through all data using realist spectacles, identifying key mechanisms and context factors that have seemed to be crucial in generating beneficial outcomes for the Foundation’s participants. In the last part, we present evidence derived from observations and perceptions of participants and key stakeholders, in support of the theoretical assumptions made in the Foundation’s PT.

### Program theory underpinning #COBW

From the #COBW policy model, we derived the following context (C), mechanism (M) and outcome (O) as building blocks of the Foundation’s PT: *Through unconstrained and inclusive activities in which people participate voluntary (C1), likeminded or similarly backgrounded peers have the opportunity to meet, to share fun and unconditioned time (M1), making it possible for participants to gain self-confidence and trust in others (O1). Such context, wherein participants feel safe and experience a sense of belonging (C2), and in which social partners collaborate in a larger network (C2), allows the participants to take up some engagement and responsibility within the team, and later on, within the Foundation and the community (M2).* As such, a learning context is shaped for the participants to build life skills such as social skills and basic work skills, and to gain a sense of usefulness (O2). This program theory is illustrated in Fig. 1.

### Key context factors

In what follows, we describe some of the necessary context factors that make SFD a successful tool to promote health. Some of these elements are at the same time (initial or intermediate) outcomes of the program’s key mechanisms and facilitating context factors for key mechanisms that may be triggered later on in the program, when necessary conditions are met - these elements are identified as (O&C).

#### Unconstrained & inclusive activities (C)

Participants of the Foundation’s activities speak of a spontaneous, fun and respectful atmosphere. They consider the activities an ideal place to ventilate, to lose frustrations, to make contact, or even friends, and to grow self-confidence.

*It does not matter whether you can play football or not. The way that we play football, makes everyone have fun, and relax. (…) Then it is fun to just empty the head a bit through football, and the social happening matters as well. (J).*

#### Voluntary participation (C)

For the Foundation it matters that participants come because they want to come, not because they are obliged. Participants are recruited by different social partners (e.g. social welfare council, the psychiatric hospitals or outreach teams…), proposing the Foundation’s activities on a voluntary basis. Data support the idea that the Foundation succeeds in motivating its participants in the long run. E.g. when participant K came to play for the first time in the team, one of the psychiatric nurses from the social network said: *“This is just a try, most likely there will be no next time.”* Yet, participant K kept on coming back. Something similar was worded in an interview with another participant: *It was new to me and I wanted to try. But I never thought I would stay this long. (I).*

#### KAA gent trade mark (C)

Many of the participants are big fans of the KAA Gent first division football team. KAA Gent is known as a football club proud of its supporters, with attention for the common man, woman or child in the street. This makes their supporters and the citizens of Ghent, football fan or not, as proud of their club as the club is of its fans. We observed the club logo on the sports outfit of the Foundation’s staff working like a magnet: children in the street shout the club’s name and play with the Foundation’s volunteers, curious parents and neighbors come to see what’s happening. Community activities organized by the Foundation are very popular events. All want to be part of the club that presents itself as one big family.

#### Sense of safety: no pressure to succeed (O&C)

Observation and interview data provided evidence for the accessible and safe environment created by the Foundation. Sense of safety is at the same time an initial outcome of the Foundation’s activities (as experienced by its participants) and a necessary context factor for further outcome.

*There is less pressure to perform. (I).*

*Everyone has his own story. And his own experience. And the moment of training (…) is a moment of letting all that go. And not really being occupied with all that. (H).*

#### Positive relation with coach (O&C)

A constructive relation with the coach being an initial output of crucial importance to further realization of the Foundation’s goals, it is essential to find proof of such relation in the interviews with participants. Although not always mentioned explicitly in interviews, evidence was found at many occasions, including observations of the activities.

*Yes, it is the best that happened to me. That I met [the coach]. (…) In the beginning we did not match. I was not always good or safe… But after a couple of months we started really talking. And at one point I said I could not go on like that. And since that moment we have continued growing. And we became friends. (B).*

*It is important to know that the coach accepts you, knows how you are with your limitations. Also important is the fact that the coach strives for equal participation in games, and does not let you sit at the sideline all the time. (I).*

#### Self-awareness & self-confidence (O&C)

In the voice and the attitude of most respondents, you can hear realization, consciousness of the length of the path they have walked. Self-awareness is not only an initial outcome of the Foundation’s activities but also a necessary condition for further personal development and wellbeing.

*[I smoke] 1 package a day. Sometimes that does not disturb you, and you’re not really occupied with it. Football makes you lose your breath, so you think about it. (E).*

The Foundation stimulates its participants to share their life stories, and as such raise awareness about issues as poverty and addiction. Doing so, participants themselves become more and more aware about their strengths, their vulnerabilities, the chances they missed, those they can or want to grab, and so forth.

*From the homeless team, I started to grow further. I started to trust myself, to grow, my uncertainties started to go away, the doubts about myself. It [the project] really drew me up. But I had enormous dells that pulled me down again. Because I made the same mistakes again. Yet, I’ve learned from that and (…) that is what makes me strong now. To learn from your own mistakes to be able to face the future positively. (A).*

All respondents come with examples of how the Foundation’s activities reinforced, in direct or indirect manner, their self-confidence.

*The coach taught me to first bring confidence in my game, and to then build towards confidence in myself, and finally trust in others, the world outside. (A).*

#### Social cohesion and sense of belonging (O&C)

Regularly, the Foundation organizes activities other than football. E.g. the Belgian Homeless Cup brings participants together with peers from all over the country in a two-days meeting: participants stay in the same accommodation and have plenty of opportunities to discuss, watch a theatre show together, go visit a village etcetera. The Foundation organizes shared lunches or dinners. Apart from the necessity, for many participants, to have a decent meal, this also serves social cohesion, since eating together is a social event in every culture.

*Yes, we are quite attached to one another. There are many friends. Two weeks ago, I went to paint, clean and organize the whole house of B. [fellow player]. (…) I invite a lot of people to come for diner at my place, for otherwise I’m just alone. (…) It is more than just sports, that’s right. (D).*

The constructive group dynamics create a powerful sense of belonging among the participants who are used to various experiences of loneliness and social exclusion. An additional facilitating context factor is the example given by the Foundation itself of treating all as part of the team, and welcoming with open arms its participants, no matter where they are in their personal trajectory: *It is one warm group, whatever happens, you stay welcome. And that is the most important for me. I think for many, yes. (C).*

#### Social partners (C)

During the training, social partners take turning roles to be present. For most participants, their presence is an important context factor.

*Yes, it does [matter that partners, such as psychiatric nurses, are present during training]. For when you are having a difficult time, you can go sit with them for a while. (I).*

*There are people who don’t dare to go [talk to the social partners]. You have to push them a little, and sometimes the coach accompanies them. Yes, once you have that [network of social partners and follow-up of participants], the rest follows automatically. (B).*

### Evidence in support of the Foundation’s PT

In the last part of the results section, we examine whether in the case of the KAA Gent Foundation unconstrained, fun and inclusive activities indeed promote meeting between likeminded people, and as such enhance self-confidence and trust, shaping a context ideal for learning life skills, including social skills, emotional skills and basic work skills.

### *Lifting barriers to get participants to play, and to stay*

Respondents confirm at many occasions that they come to the activities primarily to have fun and be able to let go of things. All mention the additional benefices (improved social contact, emotional regulation, social skills, etcetera) though, albeit in a secondary time. This confirms the existence of one of the most efficient mechanisms taking place during the Foundation’s activities: ‘learning by fun’.

*Just to have a pleasant time (…) Just the feeling, during the training, to be gone for a while, two hours away from society, from daily sorrow (…). It is distraction, most look very much forward to that time. It is that moment of the week, and there they are. (A).*

Confirmed by all respondents is the ventilating and relaxing effect of sports, especially when coach and fellow participants put the focus on fun, and not on sportive results.

*Sporting empties the head a little. (…) You can let go of things that you struggle with, and then there is room for other things. (I).*

Sport is for many an easier access to therapeutic work. Especially team sports is considered a great springboard to practicing social and emotional skills. Although football may not be the most accessible of the team sports, as one of the respondents mention: *The people that I try to convince to come with me often react with ‘oh football, that is nothing for me’. While these people do participate when it is badminton, for example. (I).*

However, the manner in which the training sessions are organized, motivates also those who have never touched a football before. It is different to regular football clubs, where focus is on result instead of fun, and there is “*too little place to have a good laugh, or to be allowed to make a mistake” (G).* The fact that *“it does not matter that much whether you can play football or not” (I)*, is for some respondents an important factor to start (and continue) to come to this group activity.

At the one hand, the Foundation actively recruits participants from socially vulnerable groups, at the other hand, it tries to lift financial barriers in order for youth from all social groups to be able to play in the local football club: *The Foundation works with children living in poverty. There are almost no financial barriers left for parents (…): kids receive sports outfits and football baskets. (H).*

Several respondents mention the fact that the accessible and respectful environment in which the Foundation’s activities take place, makes meeting and making friends easier. The Foundation organizes its activities in a way that participants feel that it is a safe environment, in which they are not obliged to keep up to certain expectations. In this, the Foundation’s activities, although supposed to lead to social and emotional learning, are nothing like meeting with the social assistant, therapist, or employment service: *You immediately feel like in a safe zone [at training]. The same as when you enter the psychiatric hospital. They don’t ask ‘where have you been?’ (H).*

This sense of safety has to do as well with feeling accepted: *Usually, when people relapse (start again with drugs or alcohol), they are told to leave. Or, they want to collocate you. They let you go. Yes, I experienced it too. But when I told the trainer here that I would not come to the training, for I was relapsing, he said ‘definitely come!’ (C).*

### *Do people with similar background meet more easily?*

For most respondents, participation to the Foundation’s activities has indeed led to enhanced social contact or an enlarged network.

*It started out with playing [football] together once, a couple of participants being quite alright, and … Then people come back, so you create a bond with them. After a while you add them to Facebook, you do a tournament together, go for a drink afterwards… (H).*

The Foundation organizes all-inclusive activities in the community, but also activities targeting specific groups, such as people facing psychosocial problems and homeless people. Does bringing them together help them to be more socially included?

*The advantage of the Funny Buffalo’s (…) is that we all have a past in psychiatry. In some, you can see that clearly; the scarfs on their arms, their legs. In others you don’t see it that well, for it is internalized, but you do know that also those have a psychiatric history. And then you may easily feel a connection. (H).*

*[It helps to have a similar story] Because you know that the other understands you. (I).*

A similar story is not enough for a connection though. One of the respondents mentions the fact that gender plays a role as well. *[I did not build a network there.] Perhaps because they’re all men. (I)*

Also the variety in where one stands in the personal process may influence the ability to connect:

*Not everyone is as far in his or her program or therapy. That is noticeable, which makes it sometimes more difficult to get in contact. Some people are more introvert, while others are a bit too social or a bit too motivated. Which can also be a reason for not connecting. (H).*

On the question whether facing the same vulnerabilities might also be of negative influence on the personal process, a respondents confirms: *The others might drag you down when they have a difficult time. (…) That is why it is handy to have different groups of friends. When you risk to be dragged down, you can drop that group. For me, there is a group at the social work place, a group in the psychiatric hospital, and since recently, a group of friends from football. (G).*

Just like similarities in life stories and difficulties may make people feel strongly connected, peer experts may serve as a powerful example for others.

*At the one hand, I do not want to be an example for I as well have made mistakes in my life; at the other hand, I do want to be one because I want to show that it is indeed possible, that you can make it finally. (…) No matter how many books you read, it is not nearly the same as what you have done or experienced yourself. You cannot just write that in a booklet. It is something that you should be able to keep for yourself and to share with those persons that need it. (A).*

### *Trust in yourself and others as necessary condition for growth*

Many participants of the Foundation’s activities have trust issues: *The most difficult thing to change is to trust. And finally, when I have a tough time, say how I really feel. Because I have a tremendous fear … to be rejected. I always think: ‘If they would know the whole content of my backpack, they will not want to get involved with me’. In the Foundation, you get the feeling ‘to be allowed’– even when I don’t fully admit to it. (C).*

Although many respondents state it takes a while before they open up and really get in touch with other participants, most of them recognize that after a while a relation of trust is built, opening up opportunities for real connection.

*After a while there is a bond of trust so for once [you dare to speak out]. Recently I sent a message to X ‘It’s not going well’. To the assistant coach as well. And those people are effectively there for you, you know? Albeit* via *a text or a call ‘keep your head up, buddy’. Without digging too deeply. (H).*

Respondents confirm the importance of trust in oneself and the others as a condition for several life skills: *The first important step is to learn to have faith in yourself and in people. If you don’t have that, you can’t progress. (A).*

### *Building experience and skills*

Study data provide many examples of social, emotional, attitudinal and work-related skills being strengthened through participation in the Foundation’s activities.

*I used to have a lot of frustration. I did not tell anyone but the consequence was that I had more fights with the coach. Now the coach is my best friend. He taught me a lot of things to lessen my frustration. That, if I’m bothered with something, I should leave for a moment. (…) That has made me change everything in fact. (…) I used to be addicted to alcohol. Now, it is different. Even if I experience stress, I no longer start to drink. (B).*

*Engagement is important. (…) Also for the trainings you engage. Together, we do achieve some sort of goal. (K).*

When alone at home without any responsibility or activity to keep you busy, it is easy to slip into isolation, and to forget how to talk to people, how to start a conversation, how to give your opinion in a respectful manner… These social skills need a bit of practice.

*You have something to do again. On Tuesday I play football and on Thursday I prepare breakfast [a community initiative for people with little means]. Those are things you do, and it does you good. Otherwise you’re just sitting at home. (D).*

A particular social skill that the Foundation is keen on and tries to stimulate at several occasions is caring for the other.

*[We learn how to care for one another]. Yes, I’ve grown in that. [That is what the coach says] I don’t see it that much yet. But indeed, the group feeling is prior for me now, instead of the football. If we don’t win, we don’t win. Then I think: ‘Ok, we tried our best’. In the past, I would never have encouraged my team mates. Now the encouragements come all by themselves. (C).*

The most basic attitudinal skills that the Foundation seems to be working on through SFD are: 1) being engaged, e.g. coming when you are expected; 2) coming on time; 3) getting through one or two hours without smoking or drinking; 4) communicating in a respectful manner; and 5) cooperating, working together for a common goal.

A respondent compares the Foundation’s activities with a social work place:

*At the one hand there is a lot of structure, at the other hand you feel useful. In the beginning I told the responsible of the work place that it was impossible for me to be more than 15 min without nicotine. But soon I could work one and a half hour between smoking breaks. (G).*

Many respondents illustrate how this works for them on or beside the sports field as well:

*When I go play football, I won’t drink, or very little. If I would not have to go play football, I would drink something, for you have nothing to do. After training I might go for a pint, yes, but it is less (…) yes, the previous year, it was more. Now, you have to go play football, so it’s difficult to take a bottle of vodka. You have to work on your condition. So you go for a run during the week. (D).*

The fact that sports is but a pleasant pretext for other than sport-related goals is beautifully illustrated by the following quote: *(…) to collaborate more and to learn from one another. Because that is what you do. Not only playing football. You hear someone saying something that is applicable to your life (…). So you constantly learn from one another. (H).*

Wherever possible, the coach makes the link visible between skills practiced in football and their use in real life.

*The coach taught us that football consists of three things: you think about it with your head, you feel it with your heart and you do it with your feet. He says it is exactly the same ‘outside’: you take your steps with your legs, you make you decisions based on feeling, but you do think about them, whether they’re the right ones. (A).*

### *The empowering effect of responsibility and engagement*

Volunteering within the Foundation, or elsewhere, is stimulated. The Foundation considers it an opportunity to build basic social and work skills, while the participant’s main motivation to volunteer is to have an occupation and to feel useful.

*I started to do the breakfast for the social welfare council on Thursday. I got in touch* via *X, a fellow player. (…) I used to take breakfast there, now I go there to help. I have to be there at 8 am, get up at 6.30 am. It gives you strength. Afterwards I eat a sandwich there and when I get home, it is already 12 am or 1 pm. On Tuesdays there is a soup café. I got acquainted through football; you get to know people who do these things [volunteering]. (…) Perhaps I can work 2 days a week somewhere to start with. Then I have Tuesday football and Thursday the breakfast, so that makes 4 days filled. (…) You see a lot of homeless at the breakfast. It fulfills me to help there. (D).*

*I am busy 7 on 7. (…) All voluntary work. As long as I’m busy, at least I’m not in the pub. (F).*

Not all participants of the Foundation’s activities are interested in taking responsibility within the Foundation, however all are asked to engage a minimum in the community activities that the Foundation invests in, e.g. sponsor runs for charity, organizing a community gathering in deprived neighborhoods, animating children in the street, etcetera. Several respondents mention how these responsibilities, how little they may be, bring about a sense of purpose, a sense of belonging. From the data, it could be identified as one of the most powerful SFD outcomes. Many socially excluded feel a nobody because they feel they only receive, and are no longer able to do something for or have some meaning for others.

*[About why sport plus is so powerful] To let one help the other. That is important to me. (…) That is meaningful: to get a role and to mean something to someone. By doing an exercise, for example. (I).*

*I never thought I would ever again be in such position in my life. That I could still, perhaps without knowing, have some meaning for people. (…) To feel useful in life, in community … Especially that. Because many of us feel like a failure. As if we walk around here doing nothing, not belonging to society. (A)*

### *Why doesn’t it work all the time, for everyone?*

As mentioned by the participant and social partners, drop-out from the Foundation’s activities is rather exceptional. When participants do not return to the activities, the reason is often a positive one, e.g. having found a job, or having one’s life back on track and for that reason no longer having the time to participate in the Foundation’s activities. However, not all participants succeed in getting their lives back on track. Asked for possible reasons why the Foundation’s theory of change does not lead to a successful outcome in some of the participants, respondents mainly point a finger at the individual’s responsibility.

*Perseverance… Continuously doubting what you can, and what you can’t do. Keep on hanging out with the wrong persons. Not wanting to learn from your mistake. If you don’t have the motivation or the will to achieve something, it is difficult to progress. (A).*

*Some people are perhaps not ready for it. Also, everyone is different. If you’re someone who constantly wants to perform and you’re not really open for accessibility and for other people; or if you feel too good for others, or look down at others because they are a bit different, then it is possible that it does not work for you. (H).*

None of the respondents states that the project has not changed a thing for them, or has not created an improvement, how small it may be.

## Discussion

In a first step we examined whether the in the Foundation’s policy plan as optimal described context (i.e. a unconstrained and inclusive culture, a positive relation with the coach, a context in which participants feel safe and accepted) was effectively put in place. Then we looked closer into the underlying assumptions of the Foundation’s PT: could evidence be found in the data that supports this theory? The KAA Gent Foundation’s interventions can be characterized as complex seen the number and difficulty of behaviors required by those delivering and receiving the intervention, seen the different groups and organizational levels targeted by the intervention, the number and variability of outcomes and the degree of tailoring allowed [40]. One of the key questions in evaluating complex interventions is what are the active ingredients and how are they exerting their effect [40]. That is why we turned to a realist evaluation.

Data suggest that the Foundation makes efforts to effectively create the necessary conditions through all of the levels of activities. Participants confirm that the activities are accessible, that it all starts light-footed and in a welcoming, warm atmosphere. They mention they keep on receiving chances from the coach and the organization as a whole – something they consider to be different with other welfare actors. Most also confirm to be able to enlarge their social network through the Foundation’s activities. Furthermore, they consider it an experiential learning space: first they learn more about themselves, their strengths and limitations; then they learn to have trust in themselves and in others, which allows them to open up and search for help when they have a difficult time.

Some successful strategies the Foundation uses to engage its participants in SFD, include activities other than playing football, volunteering and a shared engagement in community work. The most powerful context factors in the Foundation’s success story appear to be the coach(es), the peer experts among fellow participants of the activities and the link with social partners. The opportunities given to participants to take care of one another, is a strong emancipating factor, allowing participants to grow, to practice some life skills, and to feel useful with better mental health and wellbeing as a direct consequence. In the Foundation’s policy model, the final objective is employability. It is not possible to account for employability as a final outcome, because of the complexity of both the intervention and each participant’s personal context. The Foundation is but a small radar in a complex societal network and intervenes only in a limited amount of domains. There are many other influential factors that it has no control over. Moreover, the exposition time is short (on average 2 h a week), which provides only limited possibilities for a regular practice of targeted life skills.

Nevertheless, a number of important initial and intermediate outcomes could be observed, potentially though not obligatory leading to the final outcome. Participation as such, is an essential outcome to start with. As Coalter states: ‘By its very nature sport is about participation. It is about inclusion and citizenship. Sport brings individuals and communities together, highlighting commonalties…’ [41]. Participation in the Foundation’s sport activities provides important opportunities to create relations of trust – both with the coach and with peers – and to connect with others, something isolated persons do not often have the chance to. According to our data, initial and crucial outcomes following participation, are reflection and increased self-awareness – evidence that is in line with the CATCH program theory. Also at the first level, basic skills (emotion regulation, communication, being on time, engagement, respect, remediation…) are put to practice, as such enhancing the participant’s general self-efficacy.

Several theories have confirmed the importance of perceived self-efficacy or perceived competences in building lasting, intrinsic motivation to set goals for oneself and to self-manage [42–44]. It determines ‘how long people will persevere in the face of obstacles and failure experiences, their resilience to adversity, whether their thought patterns are self-hindering or self-aiding, and how much stress and depression they experience in coping with taxing environmental demands’ [42], p.625). The Foundation applies all four strategies defined by Bandura as the pathways to strengthening people’s sense of efficacy: through reduction of people’s stress reactions and altering of their negative emotional proclivities, through mastery experiences, through provision of social models and through social persuasion [42], p.625–626). All of these strategies are equally detectable in the CATCH program theory. Perhaps the most powerful strategy of the Foundation, not only to raise its participants’ sense of efficacy but also to have them practice life skills, is modeling. Seeing people similar to oneself succeed by sustained effort, raises the beliefs of newcomers and participants less far in their personal trajectory that they too have the competences to succeed [42]. What Bandura calls social persuasion, is labeled ‘motivational coaching’ in the CATCH program theory: people receive encouragement, and their attention is drawn to their success rather than their failures. The current case study shows a tremendous impact of the coach(es) on the participants. The coach’s words of appreciation carry a lot of weight, participants turn to the coach for advice of all sorts, the coach is called in case of personal problems, and so forth. The Foundation’s coaches have proven to be strong social persuaders, as such enhancing its participants’ sense of efficacy and belief in oneself. In SFD organizations perhaps even more than in regular sport clubs, positive coaching is an efficient and required technique. Rather than focusing on what is not going well, and on elimination of undesirable behavior, e.g. alcohol consumption, a ‘positive coach’ emphasizes the promotion of various competencies, including life skills that enable participants to succeed in their living environments [45, 46].

Self-efficacy plays an influential role in health and wellbeing, because it reduces people’s stress (often linked to perceived inefficacy) and it determines people’s motivation to change their health habits: ‘whether people even consider changing their health habits; whether they enlist the motivation and perseverance needed to succeed, should they choose to do so; how well they maintain the habit changes they have achieved; their vulnerability to relapse; and their success in restoring control after a setback’ [42], p. 627). A relativizing note comes from Ryan & Deci, who have highlighted the importance of self-authored motivation in contrast to more externally controlled motivation: intrinsically motivated people are more enthusiastic and interested and have more confidence, resulting in better performance, resistance, creativity, vitality, self-esteem and general wellbeing, even for people with similar levels of self-efficacy for a certain activity [44]. In the Foundation’s program theory, voluntary participation is indeed considered a necessary context factor.

While the study data provide evidence for improved wellbeing of participants of the Foundation’s activities, health nor wellbeing are explicit outcomes in the Foundation’s PT. This gives oxygen to two ideas that could be developed in a later phase or an additional study. First, it supports the portability of the mechanisms (meeting between (isolated) peers, becoming self-aware, learning by fun, group dynamics, volunteering and building experience) to other contexts. This also means that the same mechanisms might lead to different outcomes. Secondly, it is interesting to witness how the Foundation seems to succeed in improving its participants’ wellbeing although health and wellbeing are no articulated outcomes in the Foundation’s program theory. Moreover, the Foundation is relatively tolerant and unconditioned in its approach, something which is not (and most probably cannot be) the case for formal care institutions, such as psychiatric hospitals. Improved wellbeing seems to be an important intermediate outcome when working towards a more distant outcome, such as employability (being ‘the skills and abilities that allow you to be employed’ [47]. This strengthens the idea that successful health promotion requires an approach that allows the target population to set its own goals, and to develop health agency in relation to the environment, for example through valuable interpersonal relationships [48]. At least in vulnerable populations, ‘empowering interventions’ increasing one’s power to question social health norms, have proven to be more effective in promoting health than the more traditional ‘informing’ approaches [49–51]. In this study, health and wellbeing seem to be precious side-effects of guiding people to the ability to set personal objectives and to life skills promoting self-efficacy.

The Foundation’s policy model is an ideal model; the final objective, although mentioned at the top of the pyramid, is not that all participants go through the whole trajectory and find a job in the end. The organization’s major objective is to have as many persons from the target group as possible benefiting from level one, where basic life skills are practiced that enhance one’s self-esteem and self-perceived efficacy, as such increasing one’s intrinsic and long-lasting motivation to pursue personal goals, whether they are related to health, employability or social wellbeing. An impact on employability among participants of the Foundation’s activities could not be observed, or in due case, not be attributed to the Foundation alone.

***Strengths and challenges.*** Participatory observations allowed researchers to build relationships of trust with SFD participants and stakeholders, facilitating further data collection. Researchers were experienced in qualitative research, hence their awareness of potential biases associated to such trust relationships, and their capacity to mitigate them. Regular discussion and feedback from key stakeholders, peer researchers and SFD actors external to the case study at the one hand, and a parallel interventional study in another SFD organization at the other hand, challenged the researcher’s perspectives, and kept them susceptible for differing views. Future research opportunities include the follow-up on SFD participants (e.g. cohort study), in order to observe the long-term and structural effects of SFD, such as the effect on employability, and case studies rejecting the Foundation’s PT (hence challenging the approximating CATCH theory).

## Conclusion

This study aimed at examining which conditions, necessary for a successful outcome, are put forward by the studied SFD organization in promoting social inclusion, and which are the main mechanisms through which the Foundation achieves this outcome.

Among the necessary conditions for making SFD a powerful lever for social inclusion, are the background, experience and skills of the coaches and social partners involved in the Foundation’s activities – a conclusion similar to the one of the CATCH program theory. Among the most successful mechanisms of SFD are the meeting with peers, among which some experienced ones who can be a role model for others, and the possibility to engage and take responsibility in the organization or in community. The opportunities given to participants to take care of one another, is a strong emancipating factor, allowing participants to grow, to practice life skills, and to feel useful, with better mental health and wellbeing as a direct consequence. The final objective in the Foundation’s program theory is employability, but it does not expect, nor does it push, all participants to reach that goal. Life skills are practiced at all levels of the Foundation’s program theory. Wellbeing shows to be an unintended but necessary intermediate outcome on the road to employability. This is a useful insight for practitioners and policy makers. Socially vulnerable and socially excluded persons are not easy to reach. Sport activities organized in a very accessible and (culturally) acceptable manner, are a safe and fun starting point for people from the target group to return to – as shown as well in the CATCH program theory, built on insights from international literature and various national SFD projects. From that safe starting point, SFD teams that are positively coached, can grow into a social learning lab in which many of the determinants of social exclusion can be addressed. Policy makers and project funders need to be aware that the process through which socially vulnerable persons bond with peers and with coaches, is a time-consuming, however, quintessential process if the aim is to engage the target group in a sustainable self-caring dynamics leading to personal health goal-setting.

## Supplementary information

**Additional file 1.** Overview of data collected in the case study KAA Gent Foundation

## Data Availability

The datasets generated and/or analyzed during the current study are not publicly available due to the unavailability of English translations for all of the transcripts, but are available from the corresponding author on reasonable request.
