# Supplementary material for: “To mean something to someone”: sport-for-development as a lever for social inclusion
Source: Int J Equity Health. 2020 Jan 14;19:11. doi: 10.1186/s12939-019-1119-7 (PMC6961252; doi:10.1186/s12939-019-1119-7)
Supplement: Supplementary file 1 — Additional file 1. Overview of data collected in the case study KAA Gent Foundation [file 12939_2019_1119_MOESM1_ESM.pdf]

Annex 1. Overview of data collected in the case study KAA Gent Foundation

Data sources	***Time of collection***	***Hours / N***
Documents: *Policy plan; subvention policy; year reports (2017, 2018), internal documents e.g. training curriculum of Team Buffalo*	Jan - Dec 2018	
Observations	May – July 2018	57,5 hours observation
GP	May - July 2018	5 * 1,5h training<br>1 * 24h tournament with sleep-over
GB	April - July 2018	6 * 2h training<br>2 * 4h tournament and/or team activity
Buffalo League	May 2018	2 * 2h activity
Buffalo Dance Academy	May 2018	2 * 1h activity
Semi-structured interviews		
GP	Nov - Oct 2018	N=5 (1 female, 4 male)

GB	Nov - Oct 2018	N=6 (1 female, 5 male)
Focus group: *2 participants who are also member of the steering group; 1 city sports council representative; 3 representatives from psychiatric care services; 2 sport+ coordinators*	Nov 2018	N=8
